# Work–family enrichment and kindergarten teachers’ occupational commitment: the roles of job satisfaction and emotional intelligence

**DOI:** 10.3389/fpsyg.2026.1887039

**Published:** 2026-07-07

**Authors:** Lili Zhan, Haoran Zhang, Xueli Hui, Yantao Shi, Ziqing Sheng

**Affiliations:** 1 Faculty of Education, Jiangxi Normal University, Nanchang, China; 2 School of Art Design & Media, Wuhan Huaxia University of Technology, Wuhan, China; 3 College of Preschool Education, Chongqing Youth Vocational & Technical College, Chongqing, China; 4 Faculty of Education, Guangxi Normal University, Guilin, China

**Keywords:** emotional intelligence, job satisfaction, kindergarten teachers, occupational commitment, teacher psychology, work–family enrichment

## Abstract

**Objective:**

This study examined the psychological mechanism linking work–family enrichment to occupational commitment among kindergarten teachers, with job satisfaction as a mediator and emotional intelligence as a moderator.

**Design:**

A cross-sectional survey design was used to test whether job satisfaction mediated the association between work–family enrichment and occupational commitment and whether emotional intelligence moderated the direct and indirect pathways.

**Methods:**

A questionnaire survey was conducted among 1,312 kindergarten teachers in China. Participants completed measures of work–family enrichment, job satisfaction, emotional intelligence, and occupational commitment. Data were analyzed using PROCESS Models 4 and 15 with 5,000 bootstrap samples.

**Results:**

Work–family enrichment positively predicted occupational commitment. Job satisfaction partially mediated this association, indicating that work–family enrichment was related to occupational commitment both directly and indirectly through increased job satisfaction. Emotional intelligence significantly moderated this conditional process: the association between job satisfaction and occupational commitment was stronger among teachers with higher emotional intelligence, whereas the direct association between work–family enrichment and occupational commitment was stronger among teachers with lower emotional intelligence. The conditional indirect effect through job satisfaction was stronger at higher levels of emotional intelligence.

**Conclusion:**

Work–family enrichment was associated with kindergarten teachers’ occupational commitment through both direct and indirect pathways, and this association differed according to teachers’ emotional intelligence. These findings extend resource-based explanations of occupational commitment in emotionally demanding early childhood education contexts while avoiding causal claims because of the cross-sectional design.

## 1 Background

In recent years, kindergarten teachers’ occupational commitment has received increasing scholarly attention because the quality and stability of early childhood education depend not only on teachers’ professional knowledge and pedagogical skills but also on their willingness to remain in the profession and sustain long-term occupational investment. Occupational commitment refers to teachers’ psychological attachment to, identification with, and willingness to continue working in their occupation. Previous studies have shown that occupational commitment is closely associated with professional dedication, intention to stay, and positive work-related attitudes (Aryee and Tan, 1992; Collie, 2021; Räsänen et al., 2020). For kindergarten teachers, whose work is characterized by intensive emotional labor, continuous caregiving responsibilities, and frequent parent–teacher communication, occupational commitment is particularly important for maintaining the quality, continuity, and stability of early childhood education (Huang et al., 2023; Zheng et al., 2025; Zhou and Nanakida, 2023).

Kindergarten teachers often manage multiple demands from both work and family roles. These demands may create role pressure and emotional burden, but they may also generate positive resources that can be transferred across domains (Hong et al., 2021; Li et al., 2021; Wang Y. et al., 2024). Work–family enrichment refers to the extent to which experiences, resources, and positive gains obtained in one role improve individuals’ functioning in another role (Carlson et al., 2006; Greenhaus and Powell, 2006; Heskiau and McCarthy, 2021). Unlike work–family conflict, which emphasizes role incompatibility and resource depletion, work–family enrichment highlights the positive side of the work–family interface. Skills, efficacy beliefs, positive affect, social support, and relational security derived from work or family life may help teachers cope with occupational demands and maintain positive professional attitudes (McClean et al., 2021; Wang et al., 2025; Wu et al., 2021).

Previous studies have shown that work–family enrichment is positively associated with desirable work outcomes, including job satisfaction, affective commitment, organizational citizenship behavior, and job performance (Carlson et al., 2014; Chen et al., 2024; Wayne et al., 2006). Meta-analytic evidence has further confirmed the positive association between work–family enrichment and work attitudes (Guo et al., 2024; McNall et al., 2010; Zhang et al., 2018). In early childhood education, family support and work–family boundary flexibility have also been found to facilitate enrichment experiences among preschool teachers (Peng et al., 2022; Xia et al., 2024; Yang et al., 2022). Thus, work–family enrichment may constitute an important resource pathway through which kindergarten teachers maintain positive occupational attitudes and professional investment.

Recent early childhood education studies have likewise linked work–family dynamics, emotional labor, organizational support, job satisfaction, and teacher retention-related outcomes, indicating that these mechanisms are especially salient in care-intensive teaching contexts (Carey and Sutton, 2024; Eryilmaz et al., 2025; Madigan and Kim, 2021). Research on preschool teachers also suggests that organizational support, work engagement, burnout, and turnover-related mechanisms are closely connected with teachers’ work attitudes and workforce stability (Zang and Feng, 2023; Zhao et al., 2022; Zhao et al., 2023).

However, existing research has largely focused on direct associations between work–family enrichment and positive work outcomes. Less is known about the psychological mechanisms through which work–family enrichment contributes to kindergarten teachers’ occupational commitment or the conditions under which this association becomes stronger or weaker. This gap is particularly important in emotionally demanding early childhood education contexts, where teachers must continuously mobilize both external resources and internal emotional capacities (Carey and Sutton, 2024; Li et al., 2025; Zheng et al., 2024). To address this gap, the present study constructed a moderated mediation model in which job satisfaction was examined as a mediator linking work–family enrichment to occupational commitment, and emotional intelligence was examined as a moderator of this process (Wang and Qin, 2025; Zhang et al., 2025). This model clarifies how work–family enrichment is translated into occupational commitment and when this process becomes stronger or weaker, thereby providing a theoretical basis for family-friendly management, job satisfaction enhancement, and emotional competence development in early childhood education settings.

### 1.1 Theoretical framework and work–family enrichment

The present study primarily draws on work–family enrichment theory and uses conservation of resources theory and the job demands–resources model as complementary lenses. Work–family enrichment theory provides the main explanation for how positive resources are generated and transferred across work and family roles. Conservation of resources theory is used more narrowly to explain why resource gains and resource substitution may be associated with occupational attitudes. The job demands–resources model is used to clarify how resource-rich experiences and positive work evaluations may be linked to motivational and attitudinal outcomes, particularly job satisfaction and occupational commitment, in emotionally demanding teaching contexts. This arrangement reduces theoretical redundancy by assigning each framework a distinct explanatory function.

Work–family enrichment may be positively associated with kindergarten teachers’ occupational commitment. According to work–family enrichment theory, resources generated in one role can improve individuals’ functioning in another role (Greenhaus and Powell, 2006; Heskiau and McCarthy, 2021; McClean et al., 2021). Work-to-family enrichment emphasizes that positive experiences obtained at work can benefit family life, whereas family-to-work enrichment emphasizes that support and recovery obtained from family life can benefit work functioning. Thus, this theory provides the primary basis for explaining the origin and cross-domain transfer of positive resources.

For kindergarten teachers, work–family enrichment may provide emotional support, positive affect, efficacy beliefs, interpersonal skills, and relational resources. These resources are particularly relevant because kindergarten teachers’ work involves sustained emotional expression, caregiving responsibility, patience, and frequent communication with children, parents, colleagues, and administrators (Carey and Sutton, 2024; Zhao et al., 2023; Zheng et al., 2024). When teachers obtain more positive resources across work and family roles, they may be more likely to perceive their occupation as meaningful, manageable, and worth sustaining. These perceptions may be associated with stronger psychological attachment to the profession and greater willingness to remain in the occupation. Conservation of resources theory further clarifies why cross-domain resource gains may be associated with occupational commitment. Whereas work–family enrichment theory explains where these resources come from and how they move across domains, conservation of resources theory explains the attitudinal significance of resource gain and resource preservation. According to this theory, individuals strive to obtain, protect, and accumulate valued resources, and resource gains may support positive work-related attitudes (Halbesleben et al., 2014; Hobfoll, 1989; Wu et al., 2021). From this perspective, work–family enrichment may be positively associated with occupational commitment because it provides kindergarten teachers with psychological, emotional, and relational resources that support professional attachment.

Relevant empirical studies have supported this association. Work–family enrichment has been found to be positively related to job satisfaction, affective commitment, work engagement, and other favorable work outcomes (Carlson et al., 2014; Chen et al., 2024; Guo et al., 2024). In early childhood education, family support and boundary flexibility have been shown to enhance preschool teachers’ work–family enrichment (Peng et al., 2022; Wang et al., 2023; Zang and Feng, 2023), suggesting that positive cross-domain resources may be particularly important for teachers working in emotionally demanding care professions. Based on the above theoretical and empirical evidence, this study proposes Hypothesis H1:

*H1*. Work–family enrichment is positively associated with kindergarten teachers’ occupational commitment.

Although work–family enrichment may be positively associated with occupational commitment overall, its two directional facets may not have identical associations with occupational commitment. Family-to-work enrichment may be especially important for kindergarten teachers because family support and restorative experiences can replenish emotional resources and promote better work adaptation (Chen et al., 2024; McClean et al., 2021; Wu et al., 2021). Therefore, the present study also conducted supplementary analyses of the two directional facets while using overall work–family enrichment as the focal predictor in the main model.

### 1.2 Mediating role of job satisfaction

Job satisfaction is generally defined as kindergarten teachers’ positive evaluation of their occupational experiences, professional development support, and organizational environment (Eryilmaz et al., 2025; Xiang et al., 2024; Zhao and Jeon, 2024). It reflects the extent to which teachers perceive their work as meaningful, supportive, and rewarding. For kindergarten teachers, job satisfaction is not only a general work attitude but also an important psychological state shaped by the interaction between occupational demands and available resources. Because kindergarten teaching is characterized by intensive emotional labor, continuous caregiving responsibilities, and frequent parent–teacher communication, teachers’ job satisfaction may be particularly sensitive to resources obtained from both work and family domains (Liu et al., 2023a; Xia et al., 2024; Yang et al., 2022).

Work–family enrichment may be positively associated with kindergarten teachers’ job satisfaction. Work–family enrichment theory explains this association by suggesting that skills, efficacy beliefs, positive affect, emotional support, and relational resources obtained from work or family life can be transferred across domains and improve individuals’ functioning and subjective well-being (Greenhaus and Powell, 2006; Heskiau and McCarthy, 2021; McClean et al., 2021). Conservation of resources theory further explains why these resource gains may be reflected in more favorable work evaluations: resource-gain states may help individuals preserve psychological energy, maintain positive affect, and reduce emotional depletion (Halbesleben et al., 2014; Hobfoll, 2001; Hobfoll et al., 2018). Thus, teachers with higher levels of work–family enrichment may report higher job satisfaction.

Empirical studies have provided support for this association. Previous research has shown that work–family enrichment is significantly and positively related to teachers’ job satisfaction and other positive work attitudes (Carlson et al., 2014; Chen et al., 2024; Guo et al., 2024). For example, Wei et al. (2025) and Guo et al. (2024) reported that work–family enrichment was positively associated with teachers’ job satisfaction. In the context of kindergarten teaching, enrichment experiences derived from work and family domains may be readily internalized as positive evaluations of the work situation because teachers’ daily work involves sustained emotional investment and high caregiving demands (Wang et al., 2023; Wei et al., 2025; Zang and Feng, 2023). Based on the above theoretical and empirical evidence, this study proposes Hypothesis H2a:

*H2a*. Work–family enrichment is positively associated with kindergarten teachers’ job satisfaction.

Job satisfaction may also be positively associated with kindergarten teachers’ occupational commitment. At this point, the job demands–resources model provides the most specific theoretical explanation. This model suggests that job resources and positive work experiences can activate motivational and attitudinal processes, thereby enhancing engagement, motivation, and favorable occupational attitudes (Bakker et al., 2004; Collie, 2021; Xanthopoulou et al., 2007). In the present study, job satisfaction is conceptualized as an attitudinal mechanism through which resource-rich work experiences are associated with occupational commitment.

As a positive evaluation of work, job satisfaction may help teachers internalize the value of the profession, strengthen professional identity, and increase their willingness to remain in the occupation. Meta-analytic evidence further indicates that job satisfaction is negatively associated with burnout (Lee and Ashforth, 1996; Madigan and Kim, 2021; Wang Q. et al., 2024). Teachers with lower burnout and higher satisfaction are more likely to maintain stronger occupational identification and invest effort continuously in their profession, which in turn contributes to higher occupational commitment (Huang et al., 2023; Kim and Lee, 2025; Zhao et al., 2023).

Existing empirical evidence generally supports the positive relationship between job satisfaction and occupational commitment. For example, research among nurses and teachers has shown that job satisfaction is significantly and positively related to occupational commitment (Wang et al., 2012; Zheng et al., 2025; Zhou and Nanakida, 2023). However, much of the available evidence has been drawn from non-teaching occupational groups. Whether this relationship can be generalized to kindergarten teachers remains an important empirical question because kindergarten teachers face high emotional labor demands, substantial caregiving responsibilities, and frequent interactions with children and parents. Under such conditions, job satisfaction may be particularly important for linking positive work experiences with stable professional attachment. Based on the above theoretical and empirical evidence, this study proposes Hypothesis H2b:

*H2b*. Job satisfaction is positively associated with kindergarten teachers’ occupational commitment.

In all, job satisfaction may serve as a mediating mechanism in the relationship between work–family enrichment and occupational commitment. In this pathway, the three theoretical perspectives perform different but limited explanatory functions. Work–family enrichment theory explains the generation and transfer of cross-domain resources; conservation of resources theory explains why these resource gains may support positive work evaluations; and the job demands–resources model explains why job satisfaction, as a positive work-related attitude, may be associated with stronger occupational commitment. Specifically, work–family enrichment may provide kindergarten teachers with positive affective and psychological resources, which may be associated with higher job satisfaction. Higher job satisfaction may then be associated with stronger professional attachment, occupational identification, and willingness to sustain long-term investment in the profession. Although direct empirical evidence on this specific mediating pathway among kindergarten teachers remains limited, existing studies have shown that work–family enrichment is positively associated with job satisfaction and work-related commitment, providing indirect support for this inference (Carlson et al., 2014; McNall et al., 2010; Zhang et al., 2018). Based on this, the present study hypothesizes that job satisfaction mediates the relationship between work–family enrichment and occupational commitment among kindergarten teachers.

### 1.3 Moderating role of emotional intelligence

Although work–family enrichment, job satisfaction, and occupational commitment have been widely linked to positive teacher outcomes, little is known about whether emotional intelligence moderates these relationships among kindergarten teachers. Emotional intelligence refers to the ability to perceive, understand, use, and regulate emotions in oneself and others (Salovey and Mayer, 1990; Mayer and Salovey, 1993; Mayer and Salovey, 1997; Mayer et al., 2008; Wong and Law, 2002). In kindergarten teaching, where emotional labor, caregiving, and interpersonal communication are central, emotional intelligence may serve as an important personal resource.

Drawing on conservation of resources theory, emotional intelligence may shape how teachers use and transform resources into occupational commitment (Li et al., 2024a; Wang and Qin, 2025; Zhang et al., 2025). Specifically, emotional intelligence may strengthen the job satisfaction–occupational commitment link because it helps teachers transform positive work evaluations into stable professional attachment. However, it may weaken the direct work–family enrichment–occupational commitment link because teachers with higher emotional intelligence may rely less on external cross-domain resources. Thus, emotional intelligence may play both a resource-amplifying and resource-substitution role in this model.

First, emotional intelligence may moderate the relationship between job satisfaction and occupational commitment. Job satisfaction reflects teachers’ positive evaluation of their work, but this evaluation may not automatically develop into stable occupational commitment. Conservation of resources theory suggests that positive occupational attitudes are shaped not only by contextual resources, such as job satisfaction, but also by personal psychological resources, such as emotional regulation capacity and resource reserves (Halbesleben et al., 2014; Hobfoll, 1989). Teachers with higher emotional intelligence are better able to recognize, understand, and regulate emotions in demanding interpersonal situations (Li et al., 2024a; Sun et al., 2025; Zhang et al., 2025). Therefore, when they experience higher job satisfaction, they may be more likely to transform this positive work evaluation into professional identity, occupational attachment, and sustained occupational investment. In contrast, teachers with lower emotional intelligence may have greater difficulty managing negative emotions and interpersonal pressure, which may weaken the link between job satisfaction and occupational commitment.

This mechanism is particularly relevant to kindergarten teachers because their work involves intensive emotional labor and frequent interactions with children, parents, and colleagues. In this context, emotional intelligence may strengthen the extent to which job satisfaction is translated into enduring professional attachment (Li et al., 2025; Li et al., 2024b; Wang and Qin, 2025). Based on this reasoning, this study proposes Hypothesis H3a:

*H3a*. Emotional intelligence moderates the relationship between job satisfaction and kindergarten teachers’ occupational commitment. Specifically, the positive association between job satisfaction and occupational commitment is stronger among teachers with higher emotional intelligence.

Second, emotional intelligence may moderate the direct relationship between work–family enrichment and occupational commitment through a resource-substitution mechanism. Work–family enrichment provides external cross-domain resources, such as emotional support, positive affect, efficacy beliefs, and relational resources. These resources may be especially important for teachers with lower emotional intelligence, who may have greater difficulty regulating emotions and coping with caregiving demands, parent–teacher communication, and daily emotional labor. For these teachers, work–family enrichment may play a stronger compensatory role in sustaining occupational motivation and professional attachment.

By contrast, teachers with higher emotional intelligence may already possess stronger internal emotional resources. They may be better able to maintain emotional balance, manage interpersonal demands, and sustain occupational commitment even when external enrichment resources are less salient. Thus, their occupational commitment may depend less directly on work–family enrichment. This resource-substitution logic is consistent with prior research suggesting that internal and external resources may compensate for one another in shaping occupational attitudes (Heskiau and McCarthy, 2021; McClean et al., 2021; Wu et al., 2021). Based on this reasoning, this study proposes Hypothesis H3b:

*H3b*. Emotional intelligence moderates the direct relationship between work–family enrichment and kindergarten teachers’ occupational commitment. Specifically, the positive association between work–family enrichment and occupational commitment is stronger among teachers with lower emotional intelligence.

Furthermore, because emotional intelligence may strengthen the job satisfaction–occupational commitment link, it may also moderate the indirect pathway from work–family enrichment to occupational commitment through job satisfaction. Teachers with higher emotional intelligence may be more capable of transforming job satisfaction derived from work–family enrichment into stable occupational commitment. Therefore, emotional intelligence may moderate this indirect association (Li et al., 2024b; Sun et al., 2025; Zhang et al., 2025).

### 1.4 Purpose of the study

**Figure 1:**
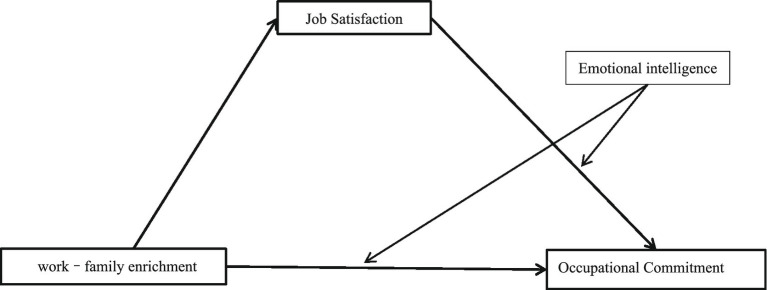
The assumed moderated mediation model.

In summary, this study examined the mediating role of job satisfaction in the relationship between work–family enrichment and kindergarten teachers’ occupational commitment, as well as the moderating role of emotional intelligence in the direct and indirect pathways. Theoretically, the study primarily draws on work–family enrichment theory and uses conservation of resources theory and the job demands–resources model as complementary explanatory lenses to clarify how cross-domain resource gains are associated with occupational commitment. Practically, the findings may help identify psychological pathways for improving kindergarten teachers’ occupational commitment and provide empirical evidence for family-friendly management, job satisfaction enhancement, and emotional competence training in early childhood education settings. The hypothesized model is presented in Figure 1.

## 2 Methods

### 2.1 Participants

This study targeted in-service kindergarten teachers in China. Using convenience sampling, teachers from public and private kindergartens were invited to complete an online questionnaire through Questionnaire Star, a widely used web-based survey platform in China. Before completing the questionnaire, participants were informed of the purpose of the study, the voluntary nature of participation, the anonymity of the survey, and the confidentiality of their responses. Only teachers who provided informed consent were allowed to proceed with the questionnaire. A total of 1,483 questionnaires were initially collected. Before the formal data analysis, all responses were screened to ensure data quality. Questionnaires were excluded if they met any of the following criteria: (a) the respondent was not an in-service kindergarten teacher; (b) the questionnaire was incomplete or contained substantial missing data; (c) the response showed obvious patterned answering, such as selecting the same option throughout the questionnaire; or (d) the completion time was unrealistically short, suggesting that the respondent may not have read the items carefully. After applying these screening criteria, 171 questionnaires were excluded. Finally, 1,312 valid questionnaires were retained for analysis, yielding a valid response rate of 88.5%.

The final sample included 1,273 female teachers (97.0%) and 39 male teachers (3.0%). Most participants (*n* = 1,259; 96.0%) majored in preschool education or a closely related education field, whereas 53 participants (4.0%) reported other majors. Regarding weekly workload, 254 teachers (19.4%) worked 40 h or fewer per week, 641 (48.9%) worked 41–50 h, 272 (20.7%) worked 51–60 h, and 145 (11.1%) worked 60 h or more. With respect to kindergarten ownership, 1,133 participants (86.4%) were employed in public kindergartens, 17 (1.3%) in collectively run kindergartens, 157 (12.0%) in non-subsidized private kindergartens, and 5 (0.4%) in collectively run kindergartens under commissioned management.

### 2.2 Procedure

This study was approved by an institutional ethics committee. Identifying details have been removed from the blinded manuscript. Prior to data collection, all participants provided written informed consent. Participants were informed that the survey was anonymous and confidential and that they could withdraw from the study at any time without penalty. The survey was administered online between April 18 and May 10, 2025. Participants completed questionnaires assessing work–family enrichment, job satisfaction, emotional intelligence, and occupational commitment. This online procedure helped maintain consistency in data collection and reduced direct contact between the research team and participants during questionnaire completion.

### 2.3 Measures

#### 2.3.1 Work–family enrichment

The Work–Family Enrichment Scale (WFES) was used to measure work–family enrichment among kindergarten teachers (Carlson et al., 2006). This scale contains 18 items and two subscales: work-to-family enrichment and family-to-work enrichment. The work-to-family enrichment subscale assesses the extent to which work experiences improve functioning in the family domain, and the family-to-work enrichment subscale assesses the extent to which family experiences improve functioning in the work domain. Example items include “Helps me to understand different viewpoints and this helps me be a better family member” and “Helps me acquire skills and this helps me be a better worker.” Each item was rated on a 5-point scale ranging from 1 = strongly disagree to 5 = strongly agree. A higher average score across these items indicates a higher level of work–family enrichment. The modified two-factor CFA model showed that the WFES had good construct validity, with *χ*^2^ = 346.440, *df* = 62, *χ*^2^/*df* = 5.588, CFI = 0.993, TLI = 0.982, RMSEA = 0.059, and SRMR = 0.011. In this study, Cronbach’s alpha coefficient for the overall scale was 0.986, and the Cronbach’s alpha coefficients for the two subscales were 0.971 and 0.981, respectively.

#### 2.3.2 Occupational commitment

The Occupational Commitment Scale was used to measure occupational commitment among kindergarten teachers (Meyer et al., 1993). This scale contains 16 items and three subscales: affective commitment, continuance commitment, and normative commitment. The affective commitment subscale assesses teachers’ emotional attachment to the occupation, the continuance commitment subscale assesses perceived costs associated with leaving the occupation, and the normative commitment subscale assesses teachers’ sense of obligation to remain in the occupation. Example items include “I would be very happy to spend the rest of my career in this occupation” and “This profession deserves my loyalty.” Each item was rated on a 5-point scale ranging from 1 = strongly disagree to 5 = strongly agree. A higher average score across these items indicates a higher level of occupational commitment. The modified three-factor CFA model showed that the Occupational Commitment Scale had good construct validity, with *χ*^2^ = 403.345, *df* = 66, *χ*^2^/*df* = 6.111, CFI = 0.983, TLI = 0.969, RMSEA = 0.062, and SRMR = 0.042. In this study, Cronbach’s alpha coefficient for the overall scale was 0.938, and the Cronbach’s alpha coefficients for the three subscales were 0.951, 0.880, and 0.909, respectively.

#### 2.3.3 Job satisfaction

Kindergarten teachers’ job satisfaction was assessed using the Chinese version of the Job Satisfaction Scale developed by Pepe et al. (2017). The scale includes nine items that assess teachers’ positive evaluations of their work experiences, professional development opportunities, and work-related relationships. A sample item is “My current job provides room for professional growth and enables me to better realize my self-worth.” Each item was rated on a 5-point scale ranging from 1 = strongly disagree to 5 = strongly agree. Higher average scores indicate higher levels of job satisfaction. The hypothesized three-factor CFA model showed good construct validity, with *χ*^2^ = 143.310, *df* = 24, *χ*^2^/*df* = 5.971, CFI = 0.991, TLI = 0.986, RMSEA = 0.062, and SRMR = 0.026. In this study, Cronbach’s alpha coefficient for the overall scale was 0.953.

#### 2.3.4 Emotional intelligence

The Wong and Law Emotional Intelligence Scale (WLEIS; Wong and Law, 2002) was used to measure emotional intelligence among kindergarten teachers. This scale contains 16 items and four subscales: self-emotion appraisal, others’ emotion appraisal, use of emotion, and regulation of emotion. The self-emotion appraisal subscale assesses teachers’ ability to understand their own emotions, the others’ emotion appraisal subscale assesses teachers’ ability to perceive others’ emotions, the use of emotion subscale assesses teachers’ ability to use emotions to facilitate performance, and the regulation of emotion subscale assesses teachers’ ability to regulate emotional states. Example items include “I usually know why I have certain feelings” and “I have strong control over my emotions.” Each item was rated on a 5-point scale ranging from 1 = strongly disagree to 5 = strongly agree. A higher average score across these items indicates a higher level of emotional intelligence. The modified four-factor CFA model showed that the WLEIS had acceptable to good construct validity, with *χ*^2^ = 620.693, *df* = 90, *χ*^2^/*df* = 6.897, CFI = 0.979, TLI = 0.972, RMSEA = 0.067, and SRMR = 0.030. In this study, Cronbach’s alpha coefficient for the overall scale was 0.963, and the Cronbach’s alpha coefficients for the four subscales were 0.946, 0.943, 0.938, and 0.958, respectively.

### 2.4 Data analysis

First, descriptive statistics and Pearson correlation analyses were conducted for all study variables to provide preliminary evidence for the hypothesized relationships (H1, H2a, and H2b). Given that all variables were collected via self-report questionnaires, Harman’s single-factor test was performed to assess potential common method bias. In addition, parcel-level confirmatory factor analysis was conducted to evaluate the discriminant validity of work–family enrichment, job satisfaction, emotional intelligence, and occupational commitment.

Second, PROCESS macro Model 4 (Hayes, 2013) was employed to test the mediating effect of job satisfaction in the relationship between work–family enrichment and occupational commitment. This model was selected because it aligns with the hypothesized indirect effect whereby work–family enrichment influences occupational commitment through job satisfaction.

Third, PROCESS macro Model 15 (Hayes, 2013) was applied to test the proposed moderated mediation model. Model 15 was chosen because it allows the moderator to simultaneously condition the mediator–outcome path and the direct effect of the independent variable on the outcome. This specification is consistent with the study’s hypotheses, in which emotional intelligence was expected to moderate both the association between job satisfaction and occupational commitment and the direct association between work–family enrichment and occupational commitment. Accordingly, Model 15 provides an appropriate analytical framework for examining whether both the direct and indirect effects vary across levels of emotional intelligence.

Statistical inference for direct, indirect, and conditional effects was conducted using bootstrapping with 5,000 resamples. Finally, robustness checks were performed by introducing demographic and work-related covariates, including gender, age, education, major, marital status, teaching experience, professional title, kindergarten type, class size, and weekly working hours.

## 3 Results

### 3.1 Common method bias and discriminant validity

The focal variables were assessed using self-report questionnaires, Harman’s single-factor test was conducted to evaluate potential common method bias. The analysis identified 13 factors with eigenvalues greater than 1, and the first factor accounted for 39.55% of the total variance, which was below the commonly used 40% threshold. This result suggested that common method bias was unlikely to substantially bias the observed associations among the study variables. A parcel-level confirmatory factor analysis was then performed to examine discriminant validity. The hypothesized four-factor model showed acceptable fit, *χ*^2^(48) = 633.91, CFI = 0.951, TLI = 0.933, RMSEA = 0.096, SRMR = 0.047, and fit the data better than the alternative models. The three-factor model, in which job satisfaction and occupational commitment were combined, showed weaker fit, *χ*^2^(51) = 1387.71, CFI = 0.889, TLI = 0.856, RMSEA = 0.141, SRMR = 0.067. The two-factor model showed poorer fit, *χ*^2^(53) = 2432.03, CFI = 0.802, TLI = 0.753, RMSEA = 0.185, SRMR = 0.081. The one-factor model fit the data poorly, *χ*^2^(54) = 4114.79, CFI = 0.661, TLI = 0.586, RMSEA = 0.240, SRMR = 0.098. These findings supported the discriminant validity of work–family enrichment, job satisfaction, emotional intelligence, and occupational commitment (see Table 1).

**Table 1:** Results of confirmatory factor analyses for discriminant validity.

Model	*χ* ^2^	*df*	CFI	TLI	RMSEA	SRMR	Interpretation
Four-factor model	633.91	48	0.951	0.933	0.096	0.047	Hypothesized model
Three-factor model: JS and OC combined	1387.71	51	0.889	0.856	0.141	0.067	Weaker fit
Two-factor model	2432.03	53	0.802	0.753	0.185	0.081	Poorer fit
One-factor model	4114.79	54	0.661	0.586	0.24	0.098	Poor fit

WFE, work–family enrichment; JS, job satisfaction; EI, emotional intelligence; OC, occupational commitment. The four-factor model included WFE, JS, EI, and OC as separate latent constructs. Poor fit.

### 3.2 Descriptive statistics and correlations

Statistical inference for direct, indirect, and conditional effects was conducted using bootstrapping with 5,000 resamples. The data were cross-sectional and based on self-report measures, all mediation and moderated mediation results were interpreted as conditional associations rather than evidence of causal processes. Finally, robustness checks were performed by introducing demographic and work-related covariates, including gender, age, education, major, marital status, teaching experience, professional title, kindergarten type, class size, and weekly working hours. Supplementary analyses were conducted to examine the two directional facets of work–family enrichment. Because these analyses were not central to the main model, detailed results are reported in Appendix A (see Table 2).

**Table 2:** Bivariate correlations among study variables.

Variable	*M*	*D*	1	2	3	4
Work–family enrichment	4.147	0.658	1			
Occupational commitment	3.748	0.616	0.456**	1		
Job satisfaction	3.946	0.605	0.570**	0.597**	1	
Emotional intelligence	3.973	0.527	0.549**	0.582**	0.618**	1

*p < 0.05, **p < 0.01, ***p < 0.001.

### 3.3 Mediating role of job satisfaction

PROCESS Model 4 (Hayes, 2013) was used to test the mediating role of job satisfaction. As shown in Table 3 (seeing Figure 1), work–family enrichment was positively associated with job satisfaction (B = 0.570, SE = 0.023, 95% CI [0.525, 0.614], *p* < 0.001). Job satisfaction was positively associated with occupational commitment after controlling for work–family enrichment (B = 0.499, SE = 0.027, 95% CI [0.447, 0.552], *p* < 0.001). Work–family enrichment remained positively associated with occupational commitment after job satisfaction was included in the model (B = 0.171, SE = 0.027, 95% CI [0.118, 0.223], *p* < 0.001).

**Table 3:** Testing the mediation effect of work–family enrichment on occupational commitment via job satisfaction.

Variables	Job satisfaction				Occupational commitment				Occupational commitment			
	B	SE	LLCI	ULCI	B	SE	LLCI	ULCI	B	SE	LLCI	ULCI
Work–family enrichment	0.569	0.023	0.525	0.614	0.171	0.027	0.118	0.223	0.456	0.025	0.407	0.504
Job satisfaction					0.499	0.027	0.447	0.552				
*R^2^*	0.325				0.376				0.208			
*F*	630.115^***^				394.362^***^				343.203^***^			

B values are unstandardized coefficients. SE, standard error; LLCI, lower limit of the 95% confidence interval; ULCI, upper limit of the 95% confidence interval. Each column is a regression model that predicts the criterion at the top of the column. *p < 0.05, ***p < 0.001.

The bootstrap analysis based on 5,000 resamples indicated a significant indirect effect of work–family enrichment on occupational commitment through job satisfaction (indirect effect = 0.285, Boot SE = 0.030, 95% Boot CI [0.226, 0.343]). Because the direct effect remained significant, job satisfaction partially mediated the association between work–family enrichment and occupational commitment. The indirect effect accounted for approximately 62.47% of the total effect. These results supported H2a, H2b, and the proposed mediating role of job satisfaction.

### 3.4 Moderating role of emotional intelligence

**Figure 2:**
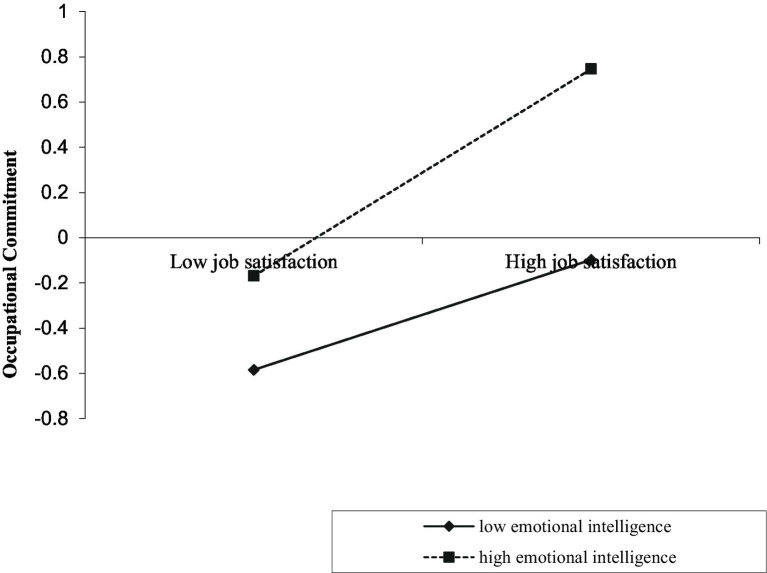
Emotional intelligence moderates the indirect relationship between job satisfaction and occupational commitment.

PROCESS Model 15 (Hayes, 2013) was used to examine whether emotional intelligence moderated the direct and indirect pathways linking work–family enrichment, job satisfaction, and occupational commitment. As shown in Table 4, job satisfaction was positively associated with occupational commitment (B = 0.351, *p* < 0.001), and the job satisfaction × emotional intelligence interaction was significant (*B* = 0.107, *p* < 0.001). Simple slope analyses showed that the association between job satisfaction and occupational commitment was stronger at high emotional intelligence (*B*simple = 0.458, *t* = 13.431, *p* < 0.001) than at low emotional intelligence (*B*simple = 0.243, *t* = 6.954, *p* < 0.001), supporting H3a (seeing Figure 2).

**Table 4:** Testing the moderated mediation effect of work–family enrichment on occupational commitment.

Variables	Model (occupational commitment)			
	B	*SE*	*LLCI*	*ULCI*
Job satisfaction	0.351	0.028	0.296	0.406
Emotional intelligence	0.314	0.028	0.261	0.368
Job satisfaction*emotional intelligence	0.107	0.020	0.067	0.146
Work–family enrichment	0.076	0.026	0.025	0.128
Work–family enrichment*emotional intelligence	−0.072	0.019	−0.111	−0.034
*R* ^2^	0.445			
*F*	209.657***			

*B* values are unstandardized coefficients. SE, standard error; LLCI, lower limit of the 95% confidence interval; ULCI, upper limit of the 95% confidence interval. ***p < 0.001.

**Figure 3:**
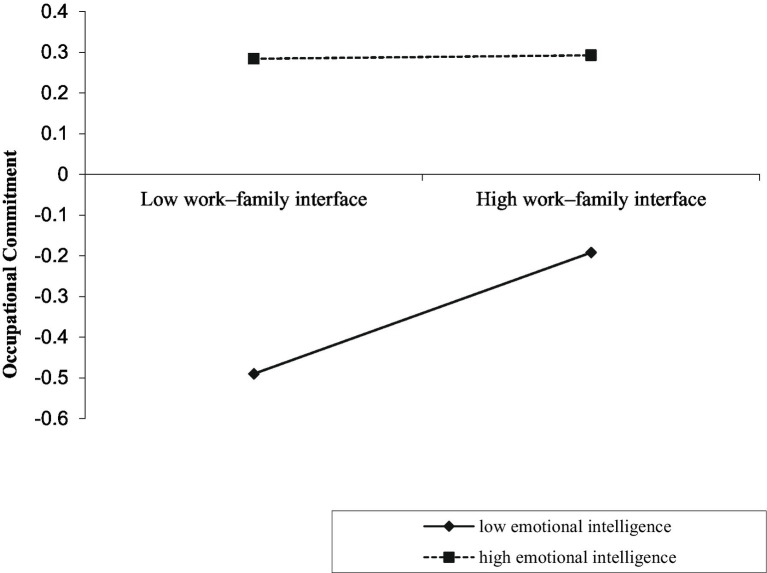
Emotional intelligence moderates the indirect relationship between work–family enrichment and occupational commitment.

Work–family enrichment was positively associated with occupational commitment (B = 0.077, *p* = 0.004, 95% CI [0.025, 0.128]). The work–family enrichment × emotional intelligence interaction was significant and negative (B = −0.072, *p* < 0.001). Simple slope analyses showed that work–family enrichment was significantly associated with occupational commitment at low emotional intelligence (*B*simple = 0.149, *t* = 4.674, *p* < 0.001), but not at high emotional intelligence (*B*simple = 0.004, *t* = 0.124, *p* = 0.901), supporting H3b (seeing Figure 3).

The conditional indirect effects were further examined. The indirect effect of work–family enrichment on occupational commitment through job satisfaction was significant at low emotional intelligence (effect = 0.139, Boot SE = 0.030, 95% Boot CI [0.081, 0.199]), at the mean level of emotional intelligence (effect = 0.200, Boot SE = 0.030, 95% Boot CI [0.143, 0.261]), and at high emotional intelligence (effect = 0.261, Boot SE = 0.038, 95% Boot CI [0.191, 0.341]). The index of moderated mediation was significant (index = 0.061, Boot SE = 0.016, 95% Boot CI [0.029, 0.093]), indicating that emotional intelligence conditioned the strength of the indirect association between work–family enrichment and occupational commitment through job satisfaction.

### 3.5 Robustness check

Robustness checks were conducted to determine whether the main findings remained stable after demographic and work-related characteristics were controlled. The covariates included gender, age, education, major, marital status, teaching years, professional title, kindergarten type, class size, and weekly work hours. The overall pattern of results was consistent with the main analyses.

In the mediation model, work–family enrichment remained positively associated with job satisfaction (B = 0.570, SE = 0.023, *p* < 0.001), job satisfaction remained positively associated with occupational commitment (B = 0.499, SE = 0.027, *p* < 0.001), and the direct association between work–family enrichment and occupational commitment remained significant (B = 0.171, SE = 0.027, *p* < 0.001). The indirect effect through job satisfaction also remained significant (indirect effect = 0.285, Boot SE = 0.030, 95% Boot CI [0.226, 0.343]).

In the moderated mediation model, both interaction terms remained significant. The job satisfaction × emotional intelligence interaction was positive and significant (B = 0.107, SE = 0.020, *p* < 0.001), whereas the work–family enrichment × emotional intelligence interaction was negative and significant (B = −0.072, SE = 0.019, *p* < 0.001). Taken together, these robustness checks indicated that the mediating role of job satisfaction and the moderating role of emotional intelligence were stable after demographic and work-related covariates were included.

## 4 Discussion

This study examined how work–family enrichment is associated with occupational commitment among kindergarten teachers and whether this association operates through job satisfaction and varies by emotional intelligence. The findings showed that work–family enrichment was positively associated with occupational commitment both directly and indirectly through job satisfaction. More importantly, emotional intelligence showed an asymmetric moderating pattern: it strengthened the job satisfaction–occupational commitment pathway but weakened the direct work–family enrichment–occupational commitment pathway. This pattern provides a more differentiated understanding of how external cross-domain resources and internal emotional resources jointly relate to teachers’ occupational commitment in early childhood education.

### 4.1 Work–family enrichment and occupational commitment

The positive association between work–family enrichment and occupational commitment extends work–family enrichment research from general work attitudes to profession-specific commitment. Previous studies have mainly focused on job satisfaction, affective commitment, work engagement, and turnover-related attitudes (Carlson et al., 2014; McNall et al., 2010; Zhang et al., 2018). By examining occupational commitment, this study shifts attention from teachers’ evaluations of a specific kindergarten or organization to their attachment to the teaching profession itself. This distinction is important in early childhood education, where workforce stability depends not only on organizational retention but also on whether teachers regard kindergarten teaching as a meaningful and sustainable career (Madigan and Kim, 2021; Zhao et al., 2022; Zhou and Nanakida, 2023).

This finding suggests that kindergarten teachers’ occupational commitment is shaped not only by professional responsibility, institutional support, or personal dedication, but also by positive resource circulation between work and family roles. Work–family enrichment theory explains that resources generated in one role can improve experiences and outcomes in another role through instrumental and affective pathways (Greenhaus and Powell, 2006; McClean et al., 2021; Wu et al., 2021). For kindergarten teachers, emotional support, efficacy beliefs, positive affect, interpersonal skills, and relational security may help them manage caregiving demands, emotional labor, classroom management, and parent–teacher communication, thereby making the profession more manageable, meaningful, and sustainable.

This result also extends conservation of resources theory by framing occupational commitment as a resource-based professional attitude. Because individuals seek to obtain, retain, and protect valued resources, resource gains may support adaptive functioning under demanding conditions (Halbesleben et al., 2014; Hobfoll, 1989; Hobfoll et al., 2018). From this perspective, kindergarten teachers’ occupational commitment may partly depend on their ability to obtain, transfer, and preserve psychological, emotional, and relational resources across roles.

The supplementary analysis of the two directional facets of work–family enrichment should be interpreted cautiously. The negative unique coefficient for work-to-family enrichment appeared only after family-to-work enrichment was simultaneously controlled, and the two facets were highly correlated. Therefore, this result is better understood as a residualized statistical effect rather than evidence that work-to-family enrichment is harmful. This nuance supports the use of overall work–family enrichment as the focal construct in the main model.

### 4.2 Job satisfaction as an attitudinal mechanism

Job satisfaction partially mediated the association between work–family enrichment and occupational commitment, indicating an attitudinal pathway through which cross-domain resources are linked to kindergarten teachers’ professional attachment. Although work–family enrichment has been widely associated with positive work outcomes, less is known about how it contributes to occupational commitment in early childhood education (Carlson et al., 2014; Chen et al., 2024; McNall et al., 2010).

The positive association between work–family enrichment and job satisfaction suggests that resources generated across work and family roles may be internalized as favorable evaluations of daily work. This mechanism is especially relevant for kindergarten teachers because their job satisfaction is closely tied to emotional and relational experiences. Support, positive affect, efficacy, and recovery from work and family roles may help teachers feel less depleted and view their work as more meaningful, supportive, and rewarding (Collie, 2021; Wang et al., 2023; Wei et al., 2025; Zang and Feng, 2023).

Job satisfaction, in turn, was positively associated with occupational commitment. In kindergarten teaching, emotional labor and caregiving responsibilities may be experienced either as meaningful professional engagement or as an exhausting burden. Higher job satisfaction may help teachers transform positive daily work experiences into stronger professional attachment and sustained occupational investment. This interpretation is consistent with evidence linking job satisfaction to teacher commitment, retention-related attitudes, and positive occupational functioning (Eryilmaz et al., 2025; Huang et al., 2023; Madigan and Kim, 2021; Zheng et al., 2025).

The partial mediation also suggests that job satisfaction is an important but not exhaustive mechanism. Work–family enrichment may also contribute to occupational commitment through reduced burnout, enhanced work engagement, stronger professional identity, perceived organizational support, or psychological resilience. This view is consistent with the job demands–resources model, which suggests that job and personal resources support motivational processes and positive occupational outcomes under demanding work conditions (Bakker and Demerouti, 2017; Demerouti et al., 2001).

In all, the mediation result clarifies the complementary roles of the theoretical frameworks. Work–family enrichment theory explains cross-role resource transfer (Greenhaus and Powell, 2006); conservation of resources theory explains the value of resource gains for preserving psychological energy and positive work evaluations (Hobfoll, 1989; Hobfoll et al., 2018); and the job demands–resources model explains how job satisfaction may link resource-rich experiences to stronger occupational commitment (Bakker and Demerouti, 2017; Demerouti et al., 2001).

### 4.3 Emotional intelligence as a resource-dependence boundary condition

The moderation findings provide the central theoretical contribution of this study. Emotional intelligence did not uniformly strengthen all positive associations. Instead, it showed an asymmetric moderating pattern: it strengthened the job satisfaction–occupational commitment pathway but weakened the direct work–family enrichment–occupational commitment pathway. This suggests that emotional intelligence should be understood as a resource-dependence boundary condition rather than simply as a uniformly beneficial personal resource.

For the job satisfaction–occupational commitment pathway, emotional intelligence functioned as a resource-amplifying factor. Although job satisfaction reflects a positive evaluation of work, it may not automatically develop into stable occupational commitment. Teachers with higher emotional intelligence may be better able to preserve positive affect, regulate frustration, manage interpersonal tension, and integrate favorable work experiences into their professional self-understanding. Thus, emotional intelligence may help transform job satisfaction into more enduring occupational attachment. This interpretation is consistent with studies linking emotional intelligence to teachers’ emotional regulation, occupational well-being, and adaptive work outcomes (Li et al., 2024a; Li et al., 2024b; Li et al., 2025; Mayer and Salovey, 1997; Mayer et al., 2008; Salovey and Mayer, 1990; Zhang et al., 2025).

By contrast, the direct work–family enrichment–occupational commitment pathway followed a resource-substitution logic. Work–family enrichment represents an external cross-domain resource, whereas emotional intelligence represents an internal emotional resource. Teachers with lower emotional intelligence may depend more on external resources from the work–family interface to manage emotional labor, parent–teacher communication, and caregiving stress. For teachers with higher emotional intelligence, stronger internal emotional regulation resources may reduce their direct dependence on work–family enrichment. This finding refines conservation of resources theory by suggesting that internal and external resources may operate additively in some pathways but substitutively in others (Halbesleben et al., 2014; Hobfoll et al., 2018).

The conditional indirect effect further supports this interpretation. The indirect association between work–family enrichment and occupational commitment through job satisfaction was stronger at higher levels of emotional intelligence. Thus, H3a and H3b are not contradictory: emotional intelligence amplified the conversion of job satisfaction into occupational commitment while reducing teachers’ direct dependence on external work–family resources.

### 4.4 Practical implications

The findings suggest that kindergarten teachers’ occupational commitment can be promoted through integrated intervention programs targeting work–family resources, job satisfaction, and emotional competence.

Firstly, kindergartens should establish work–family resource support programs. These programs may include flexible scheduling, workload monitoring, team-based duty rotation, supportive leave arrangements, and regular consultation mechanisms for teachers experiencing work–family difficulties. By facilitating resource transfer between work and family roles, such programs may help reduce emotional depletion and sustain teachers’ professional attachment in care-intensive teaching contexts (Carey and Sutton, 2024; Xia et al., 2024; Zhao and Jeon, 2024).

Secondly, teacher work experience improvement programs should be used to enhance job satisfaction. These programs may include novice teacher mentoring, peer collaboration groups, transparent evaluation procedures, professional development workshops, career planning support, and formal recognition of emotional labor. By improving perceived support, professional meaning, and growth opportunities, these interventions may strengthen teachers’ willingness to remain committed to early childhood education (Collie, 2021; Liu et al., 2023b; Xiang et al., 2024).

Thirdly, emotional competence training should be incorporated into continuous professional development. Specific modules may include emotion awareness training, emotion regulation practice, parent–teacher communication simulations, stress coping workshops, and reflective supervision based on classroom cases. Differentiated support is also important: teachers with lower emotional intelligence may benefit more from basic regulation and coping training, whereas teachers with higher emotional intelligence may benefit more from reflective practice and professional identity development programs (Li et al., 2025; Wang and Qin, 2025; Zhang et al., 2025).

### 4.5 Limitations and future directions

Several limitations should be acknowledged. First, the cross-sectional design limits causal inference, and the temporal order among work–family enrichment, job satisfaction, emotional intelligence, and occupational commitment cannot be firmly established. Future studies should use longitudinal or experimental designs.

Second, all variables were measured using self-report questionnaires. Although common method bias tests did not indicate a serious problem, self-report and social desirability biases may still exist. Future research could use multisource or time-lagged data.

Third, the sample was obtained through convenience sampling and consisted mainly of female kindergarten teachers. Future studies should include more diverse samples across regions, kindergarten types, and demographic groups.

Finally, this study focused on job satisfaction as the mediator and emotional intelligence as the moderator. Future research could examine other mechanisms, such as burnout, work engagement, professional identity, perceived organizational support, or psychological resilience, and compare additive, buffering, and substitution models of internal and external resources.

Overall, this study showed that work–family enrichment was associated with stronger occupational commitment among kindergarten teachers both directly and indirectly through job satisfaction. Emotional intelligence strengthened the job satisfaction–occupational commitment pathway but weakened the direct work–family enrichment–occupational commitment pathway. These findings suggest that emotional intelligence functions both as a personal emotional resource and as a resource-dependence boundary condition. The study contributes to resource-based explanations of kindergarten teachers’ occupational commitment and highlights the value of integrating work–family support, job satisfaction enhancement, and emotional competence development in early childhood education settings.

## Data Availability

The original contributions presented in the study are included in the article/supplementary material, further inquiries can be directed to the corresponding author.

## Appendix A

### Supplementary analyses of the directional facets of work–family enrichment

To further examine whether the two directional facets of work–family enrichment showed different associations with occupational commitment, a supplementary multiple linear regression analysis was conducted using work-to-family enrichment and family-to-work enrichment as simultaneous predictors of occupational commitment. The results showed that family-to-work enrichment was positively associated with occupational commitment (B = 0.541, SE = 0.053, *β* = 0.608, *t* = 10.108, *p* < 0.001). By contrast, when family-to-work enrichment was controlled, work-to-family enrichment was negatively associated with occupational commitment (B = −0.136, SE = 0.057, *β* = −0.144, *t* = −2.390, *p* = 0.017). Because the two facets were highly correlated (*r* = 0.915), this negative coefficient should be interpreted as a residualized suppression effect under simultaneous adjustment rather than as evidence that work-to-family enrichment is harmful in itself. The variance inflation factor for both directional predictors was 6.14, indicating elevated but not extreme multicollinearity. These findings suggest that the family-to-work direction may be more strongly and positively associated with kindergarten teachers’ occupational commitment than the work-to-family direction; however, because of the high overlap between the two facets, the overall work–family enrichment score remained the focal predictor in the main model (Table A1).

**Table A1:** Multiple linear regression analysis predicting occupational commitment from work-to-family enrichment and family-to-work enrichment.

Variable	B	SE	β	t	p
Constant	2.082	0.097	—	21.531	<0.001
Work-to-family enrichment	−0.136	0.057	−0.144	−2.390	0.017
Family-to-work enrichment	0.541	0.053	0.608	10.108	<0.001

The dependent variable was occupational commitment. B = unstandardized regression coefficient; SE = standard error; *β* = standardized regression coefficient. *p*-values are two-tailed.

## References

[ref1] Aryee S. Tan K. (1992). Antecedents and outcomes of career commitment. J. Vocat. Behav. 40, 288–305. doi: 10.1016/0001-8791(92)90052-2

[ref2] Bakker A. B. Demerouti E. (2017). Job demands–resources theory: taking stock and looking forward. J. Occup. Health Psychol. 22, 273–285. doi: 10.1037/ocp0000056, 27732008

[ref3] Bakker A. B. Demerouti E. Verbeke W. (2004). Using the job demands-resources model to predict burnout and performance. Hum. Resour. Manag. 43, 83–104. doi: 10.1002/hrm.20004

[ref4] Carey S. Sutton A. (2024). Early childhood teachers’ emotional labour: the role of job and personal resources in protecting well-being. Teach. Teach. Educ. 148:104699. doi: 10.1016/j.tate.2024.104699

[ref5] Carlson D. S. Hunter E. M. Ferguson M. Whitten D. (2014). Work–family enrichment and satisfaction: mediating processes and relative impact of originating and receiving domains. J. Manag. 40, 845–865. doi: 10.1177/0149206311414429

[ref6] Carlson D. S. Kacmar K. M. Wayne J. H. Grzywacz J. G. (2006). Measuring the positive side of the work-family interface: development and validation of a work-family enrichment scale. J. Vocat. Behav. 68, 131–164. doi: 10.1016/j.jvb.2005.02.002

[ref7] Chen Y. Zhao G. Cheng M. Y. (2024). Longitudinal associations between the rates of change in family to work enrichment, leader-member exchange, and job satisfaction. J. Vocat. Behav. 150:103986. doi: 10.1016/j.jvb.2024.103986

[ref8] Collie R. J. (2021). A multilevel examination of teachers’ occupational commitment: the roles of job resources and disruptive student behavior. Soc. Psychol. Educ. 24, 387–411. doi: 10.1007/s11218-021-09617-y

[ref9] Demerouti E. Bakker A. B. Nachreiner F. Schaufeli W. B. (2001). The job demands–resources model of burnout. J. Appl. Psychol. 86, 499–512. doi: 10.1037/0021-9010.86.3.499, 11419809

[ref10] Eryilmaz N. Kennedy A. I. Strietholt R. Johansson S. (2025). Teacher job satisfaction: international evidence on the role of school working conditions and teacher characteristics. Stud. Educ. Eval. 86:101474. doi: 10.1016/j.stueduc.2025.101474

[ref11] Greenhaus J. H. Powell G. N. (2006). When work and family are allies: a theory of work-family enrichment. Acad. Manag. Rev. 31, 72–92. doi: 10.5465/amr.2006.19379625

[ref12] Guo Y. Wang S. Rofcanin Y. Las Heras M. (2024). A meta-analytic review of family supportive supervisor behaviors (FSSBs): work-family related antecedents, outcomes, and a theory-driven comparison of two mediating mechanisms. J. Vocat. Behav. 151:103988. doi: 10.1016/j.jvb.2024.103988

[ref13] Halbesleben J. R. B. Neveu J. P. Paustian-Underdahl S. C. Westman M. (2014). Getting to the “COR”. J. Manag. 40, 1334–1364. doi: 10.1177/0149206314527130

[ref14] Hayes A. F. (2013). Introduction to Mediation, Moderation, and Conditional Process Analysis: A Regression-based Approach. New York, NY: Guilford Press.

[ref15] Heskiau R. McCarthy J. M. (2021). A work-family enrichment intervention: transferring resources across life domains. J. Appl. Psychol. 106, 1573–1585. doi: 10.1037/apl0000833, 33017156

[ref16] Hobfoll S. E. (1989). Conservation of resources: a new attempt at conceptualizing stress. Am. Psychol. 44, 513–524. doi: 10.1037/0003-066X.44.3.513, 2648906

[ref17] Hobfoll S. E. (2001). The influence of culture, community, and the nested-self in the stress process: advancing conservation of resources theory. Appl. Psychol. 50, 337–421. doi: 10.1111/1464-0597.00062

[ref18] Hobfoll S. E. Halbesleben J. Neveu J. P. Westman M. (2018). Conservation of resources in the organizational context: the reality of resources and their consequences. Annu. Rev. Organ. Psychol. Organ. Behav. 5, 103–128. doi: 10.1146/annurev-orgpsych-032117-104640

[ref19] Hong X. Liu Q. Zhang M. (2021). Dual stressors and female pre-school teachers’ job satisfaction during the COVID-19: the mediation of work-family conflict. Front. Psychol. 12:691498. doi: 10.3389/fpsyg.2021.691498, 34168602 PMC8217622

[ref20] Huang W. Zhang S. Li H. (2023). Effects of person-job fit on occupational commitment among kindergarten teachers: occupational well-being as mediator and perceived organizational support as moderator. BMC Psychol. 11:402. doi: 10.1186/s40359-023-01441-7, 37986096 PMC10658734

[ref21] Kim S. Lee J. (2025). The mediating effect of teacher commitment in the relationship between burnout and role performance of early childhood teachers. Front. Educ. 9:1474838. doi: 10.3389/feduc.2024.1474838

[ref22] Lee R. T. Ashforth B. E. (1996). A meta-analytic examination of the correlates of the three dimensions of job burnout. J. Appl. Psychol. 81, 123–133. doi: 10.1037/0021-9010.81.2.123, 8603909

[ref23] Li M. Cheng R. Liu F. (2024a). Teachers’ emotional intelligence and job satisfaction: the mediating roles of expression of naturally felt emotion and perceived teacher-student closeness. Psychol. Sch. 61, 4792–4808. doi: 10.1002/pits.23307

[ref24] Li X. Guo Y. Zhou S. (2021). Chinese preschool teachers’ income, work-family conflict, organizational commitment, and turnover intention: a serial mediation model. Child Youth Serv. Rev. 128:106005. doi: 10.1016/j.childyouth.2021.106005

[ref25] Li Y. Huang L. Naseem S. Shen Q. (2025). How emotional intelligence affects college teachers’ wellbeing in China? The mediating role of work-family support. Front. Psychol. 16:1517842. doi: 10.3389/fpsyg.2025.1517842, 40264999 PMC12013114

[ref26] Li M. Liu F. Yang C. (2024b). Teachers’ emotional intelligence and organizational commitment: a moderated mediation model of teachers’ psychological well-being and principal transformational leadership. Behav. Sci. 14:345. doi: 10.3390/bs14040345, 38667141 PMC11048059

[ref27] Liu Y. Wang W. Liu J. (2023a). Work-related use of information and communication technologies (W_ICTs) and job satisfaction of kindergarten teachers: a moderated mediation model. Acta Psychol. 237:103947. doi: 10.1016/j.actpsy.2023.103947, 37244056

[ref28] Liu Y. Yu Y. Zeng X. Li Y. (2023b). Linking preschool teachers’ pay equity and turnover intention in Chinese public kindergartens: the mediating role of perceived organizational support and job satisfaction. Sustainability 15:13258. doi: 10.3390/su151713258

[ref29] Madigan D. J. Kim L. E. (2021). Towards an understanding of teacher attrition: a meta-analysis of burnout, job satisfaction, and teachers’ intentions to quit. Teach. Teach. Educ. 105:103425. doi: 10.1016/j.tate.2021.103425

[ref30] Mayer J. D. Salovey P. (1993). The intelligence of emotional intelligence. Intelligence 17, 433–442. doi: 10.1016/0160-2896(93)90010-3

[ref31] Mayer J. D. Salovey P. (1997). “What is emotional intelligence?” in Emotional Development and Emotional Intelligence: Educational Implications, eds. Salovey P. Sluyter D. J. (New York, NY: Basic Books).

[ref32] Mayer J. D. Salovey P. Caruso D. R. (2008). Emotional intelligence: New ability or eclectic traits? Am. Psychol. 63, 503–517. doi: 10.1037/0003-066X.63.6.503, 18793038

[ref33] McClean S. T. Yim J. Courtright S. H. Dunford B. B. (2021). Transformed by the family: An episodic, attachment theory perspective on family-work enrichment and transformational leadership. J. Appl. Psychol. 106, 1848–1866. doi: 10.1037/apl0000869, 33617277

[ref34] McNall L. A. Nicklin J. M. Masuda A. D. (2010). A meta-analytic review of the consequences associated with work–family enrichment. J. Bus. Psychol. 25, 381–396. doi: 10.1007/s10869-009-9141-1

[ref35] Meyer J. P. Allen N. J. Smith C. A. (1993). Commitment to organizations and occupations: extension and test of a three-component conceptualization. J. Appl. Psychol. 78, 538–551. doi: 10.1037/0021-9010.78.4.538

[ref36] Peng Q. Lian C. Zhang L. (2022). Influence of border-keepers’ support on work-family enrichment of preschool teachers in China: the mediating role of work-family boundary flexibility. Front. Psychol. 12:752836. doi: 10.3389/fpsyg.2021.752836, 35222139 PMC8865368

[ref37] Pepe A. Addimando L. Veronese G. (2017). Measuring teacher job satisfaction: assessing invariance in the teacher job satisfaction scale (TJSS) across six countries. Eur. J. Psychol. 13, 396–416. doi: 10.5964/ejop.v13i3.1389, 28904592 PMC5590527

[ref38] Räsänen K. Pietarinen J. Pyhältö K. Soini T. Väisänen P. (2020). Why leave the teaching profession? A longitudinal approach to the prevalence and persistence of teacher turnover intentions. Soc. Psychol. Educ. 23, 837–859. doi: 10.1007/s11218-020-09567-x

[ref39] Salovey P. Mayer J. D. (1990). Emotional intelligence. Imag. Cogn. Pers. 9, 185–211. doi: 10.2190/DUGG-P24E-52WK-6CDG

[ref40] Sun S. Yan Z. Sun C. (2025). Kindergarten teachers’ emotional intelligence and surface acting: the chain mediating effects of self-efficacy and work engagement. Front. Psychol. 16:1434407. doi: 10.3389/fpsyg.2025.1434407, 39931285 PMC11808152

[ref41] Wang Q. Gao Y. Wang X. (2024). The relationships between burnout, job satisfaction, and emotion regulation among foreign language teachers: a meta-analytic review. Acta Psychol. 250:104545. doi: 10.1016/j.actpsy.2024.104545, 39476706

[ref42] Wang L. Qiao T. Wang X. Wang C. Ye P. (2025). The impact of work–family conflict on early childhood teachers’ occupational well-being: the chain mediating role of psychological empowerment and job crafting. Front. Public Health 12:1513514. doi: 10.3389/fpubh.2024.1513514, 39845653 PMC11753349

[ref43] Wang D. Qin J. (2025). Research development of teachers’ emotional intelligence in the 21st century: a bibliometric analysis. Acta Psychol. 257:105067. doi: 10.1016/j.actpsy.2025.105067, 40398267

[ref44] Wang L. Tao H. Ellenbecker C. H. Liu X. (2012). Job satisfaction, occupational commitment and intent to stay among Chinese nurses: a cross-sectional questionnaire survey. J. Adv. Nurs. 68, 539–549. doi: 10.1111/j.1365-2648.2011.05755.x, 21722170

[ref45] Wang Y. Xia Q. Yue H. Teng W. (2024). Chinese rural kindergarten teachers’ work-family conflict and their turnover intention: the role of emotional exhaustion and professional identity. Behav. Sci. 14:597. doi: 10.3390/bs14070597, 39062420 PMC11273554

[ref46] Wang Y. Xia Q. Yue H. Yu R. Zhang W. Li J. . (2023). The relationship between work–family conflict and job satisfaction for preschool teachers in rural China: a moderated mediation model. Front. Public Health 11:1236713. doi: 10.3389/fpubh.2023.1236713, 38125845 PMC10731268

[ref47] Wayne J. H. Randel A. E. Stevens J. (2006). The role of identity and work-family support in work-family enrichment and its work-related consequences. J. Vocat. Behav. 69, 445–461. doi: 10.1016/j.jvb.2006.07.002

[ref48] Wei S. H. Wang Q. Song S. (2025). The relationship between pro-family work support and job satisfaction among primary and secondary school teachers: the mediating role of work-to-family conflict, work-to-family enrichment and work engagement. Curr. Psychol. 44, 4818–4830. doi: 10.1007/s12144-025-07545-0

[ref49] Wong C. S. Law K. S. (2002). The effects of leader and follower emotional intelligence on performance and attitude. Leadersh. Q. 13, 243–274. doi: 10.1016/S1048-9843(02)00099-1

[ref50] Wu C. Hunter E. M. Sublett L. W. (2021). Gaining affective resources for work-family enrichment: a multisource experience sampling study of micro-role transitions. J. Vocat. Behav. 125:103541. doi: 10.1016/j.jvb.2021.103541

[ref51] Xanthopoulou D. Bakker A. B. Demerouti E. Schaufeli W. B. (2007). The role of personal resources in the job demands-resources model. Int. J. Stress. Manag. 14, 121–141. doi: 10.1037/1072-5245.14.2.121

[ref52] Xia W. Fan Y. Bai J. Zhang Q. Wen Y. (2024). The relationship between organizational climate and job satisfaction of kindergarten teachers: a chain mediation model of occupational stress and emotional labor. Front. Psychol. 15:1373892. doi: 10.3389/fpsyg.2024.1373892, 38863665 PMC11165699

[ref53] Xiang X. Wei Y. Lei Y. Li W. He X. (2024). Impact of psychological empowerment on job satisfaction among preschool teachers: mediating role of professional identity. Humanit. Soc. Sci. Commun. 11:1175. doi: 10.1057/s41599-024-03706-x

[ref54] Yang Y. Lu X. Ban Y. Sun J. (2022). Social support and job satisfaction in kindergarten teachers: the mediating role of coping styles. Front. Psychol. 13:809272. doi: 10.3389/fpsyg.2022.809272, 35360644 PMC8963865

[ref55] Zang L. Feng Y. (2023). Relationship between job satisfaction and work engagement in Chinese kindergarten teachers: vocational delay of gratification as a mediator. Front. Psychol. 14:1114519. doi: 10.3389/fpsyg.2023.1114519, 36910796 PMC9997526

[ref56] Zhang Z. Li Y. Wang Y. An X. (2025). The influence of 8,786 Western China kindergarten teachers’ emotional intelligence on work engagement. Front. Psychol. 16:1542911. doi: 10.3389/fpsyg.2025.1542911, 40201749 PMC11977667

[ref57] Zhang Y. Xu S. Jin J. Ford M. T. (2018). The within and cross-domain effects of work-family enrichment: a meta-analysis. J. Vocat. Behav. 104, 210–227. doi: 10.1016/j.jvb.2017.11.003

[ref58] Zhao N. Huo M. Van Den Noortgate W. (2023). Exploring burnout among preschool teachers in rural China: a job demands-resources model perspective. Front. Psychol. 14:1253774. doi: 10.3389/fpsyg.2023.1253774, 37885751 PMC10598671

[ref59] Zhao X. Jeon L. (2024). Examining the associations between teacher job satisfaction, workplace climate, and well-being resources within head start programs. Early Educ. Dev. 35, 933–949. doi: 10.1080/10409289.2023.2221765

[ref60] Zhao Y. Lu Z. Cheng X. Li J. (2022). The effect of organizational trust on turnover intention of rural kindergarten teachers: the mediating role of teaching efficacy and job satisfaction. Int. J. Environ. Res. Public Health 19:12403. doi: 10.3390/ijerph191912403, 36231702 PMC9566009

[ref61] Zheng X. Luo Y. Tang J. (2024). Kindergarten teachers’ emotional labor and occupational well-being: how do different interpersonal relationships matter? Early Educ. Dev. 35, 1862–1876. doi: 10.1080/10409289.2024.2360871

[ref62] Zheng Y. Wang X. Yang L. (2025). Explore the correlations of occupational commitment, psychological resilience, job satisfaction, and burnout in kindergarten teachers’ turnover intentions in rural China. Front. Psychol. 16:1605831. doi: 10.3389/fpsyg.2025.1605831, 40657573 PMC12247534

[ref63] Zhou Y. F. Nanakida A. (2023). Job satisfaction and self-efficacy of in-service early childhood teachers in the post-COVID-19 pandemic era. Humanit. Soc. Sci. Commun. 10:721. doi: 10.1057/s41599-023-02174-z

